# Effect of Clazosentan in Patients with Aneurysmal Subarachnoid Hemorrhage: A Meta-Analysis of Randomized Controlled Trials

**DOI:** 10.1371/journal.pone.0047778

**Published:** 2012-10-17

**Authors:** Xiang Wang, Yi-Ming Li, Wei-Qing Li, Cheng-Guang Huang, Yi-Cheng Lu, Li-Jun Hou

**Affiliations:** 1 Department of Neurosurgery, Second Military Medical University, Changzheng Hospital, Shanghai, China; 2 Department of Pathology, Second Military Medical University, Changzheng Hospital, Shanghai, China; St Michael's Hospital, University of Toronto, Canada

## Abstract

**Background:**

Cerebral vasospasm is the most important potentially treatable cause of mortality and morbidity following aneurysmal subarachnoid hemorrhage (aSAH). Clazosentan, a selective endothelinreceptor antagonist, has been suggested to help reduce the incidence of vasospasm in patients with aSAH. However, the results were controversial in previous trials. This meta-analysis attempts to assess the effect of clazosentan in patients with aSAH.

**Methodology/Principal Findings:**

We systematically searched Pubmed, Embase, and the Cochrane Library from their inception until June, 2012. All randomized controlled trials (RCTs) related to the effect of clazosentan in aSAH were included. The primary outcomes included the incidence of angiographic vasospasm, new cerebral infarction (NCI), delayed ischemic neurological deficits (DIND), and vasospasm-related morbidity/mortality (M/M); the second outcomes included the occurrence of rescue therapy, all-cause-mortality, and poor outcome. 4 RCTs were included with a total of 2156 patients. The risk of angiographic vasospasm (relative risk [RR] = 0.58; 95% CI, 0.48 to 0.71), DIND (RR = 0.76; 95% CI, 0.62 to 0.92), and vasospasm-related M/M (RR = 0.80; 95% CI, 0.67 to 0.96) were statistically significantly reduced in the clazosentan group. Patients treated with clazosentan had a reduced occurrence of rescue therapy (RR = 0.62; 95% CI, 0.49 to 0.79). However, no statistically significant effects were observed in NCI (RR = 0.74; 95% CI, 0.52 to 1.04), mortality (RR = 1.03; 95% CI, 0.71 to 1.49), and poor outcome (RR = 1.12; 95% CI, 0.96 to 1.30).

**Conclusions/Significance:**

Our pooling data supports that clazosentan is probably effective in preventing the occurrence of angiographic vasospasm, vasospasm-related DIND, vasospasm related M/M, and rescue therapy. However, no evidence lends significant supports to the benefits of clazosentan in decreasing the occurrence of NCI, mortality or improving the functional outcome.

## Introduction

Despite significant advances in treatment of aneurysmal subarachnoid hemorrhage (aSAH), the outcome remains poor.At the very least, a quarter patients with aSAH died, and roughly half of the survivors had persistent neurological deficits [1]. Cerebral vasospasm is the most important potentially treatable cause of mortality and morbidity following aSAH [2]. It is recognized as the primary cause of delayed cerebral ischemia (DCI) and the consequent delayed ischemic neurological deficits (DIND) [2].

So far, the pathogenesis of cerebral vasospasm remains incompletely understood. Current management of cerebral vasospasm, including calcium channel antagonists, statins, magnesium sulphate, triple-H therapy (hypervolemia, hypertension, and hemodilution), and rescue therapy with angioplasty and intra-arterial infusion of vasodilators, still shows variable effectiveness [1].

Recently, it has been indicated that endothelin-1 (ET) plays a critical role in the pathogenesis of vasospasm [3]. Clazosentan, the first nonpeptide selective ET_A_ receptor antagonist, was thought to be a “magic bullet” to inhibit endothelin-mediating vasospasm [2]. Some trials aboutthe effect of clazosentan in aSAH have been conducted but with inconsistent conclusions. For instance, some investigators suggested that clazosentan decreased the severity and the incidence of vasospasm [4], [5], or the vasospasm-related morbidity/mortality (M/M) [5], [6], while others argued against that [7]. Thus, we performed this meta-analysis of clazosentan treatment after aSAH, aiming to determine the positive effects and associated adverse effect (AE) of clazosentanintreating aSAH.

## Methods

### Search Strategy

The overview of RCTs was conducted in accordance with the Preferred Reporting Items for Systematic Reviews and Meta-analysis (PRISMA) statement [8]. We systematically searched Pubmed, Embase, and the Cochrane Library from their inception until June 2012 without language restriction, and identified all RCTs related to the effects of clazosentan in aSAH. We used the following search keywords: “clazosentan”, “intracranial aneurysm”, “subarachnoid hemorrhage”,“vasospasm”, and “randomized controlled trial”. Additionally, we manually searched the references of selective papers to identify additional potentially eligible studies.

**Figure 1:**
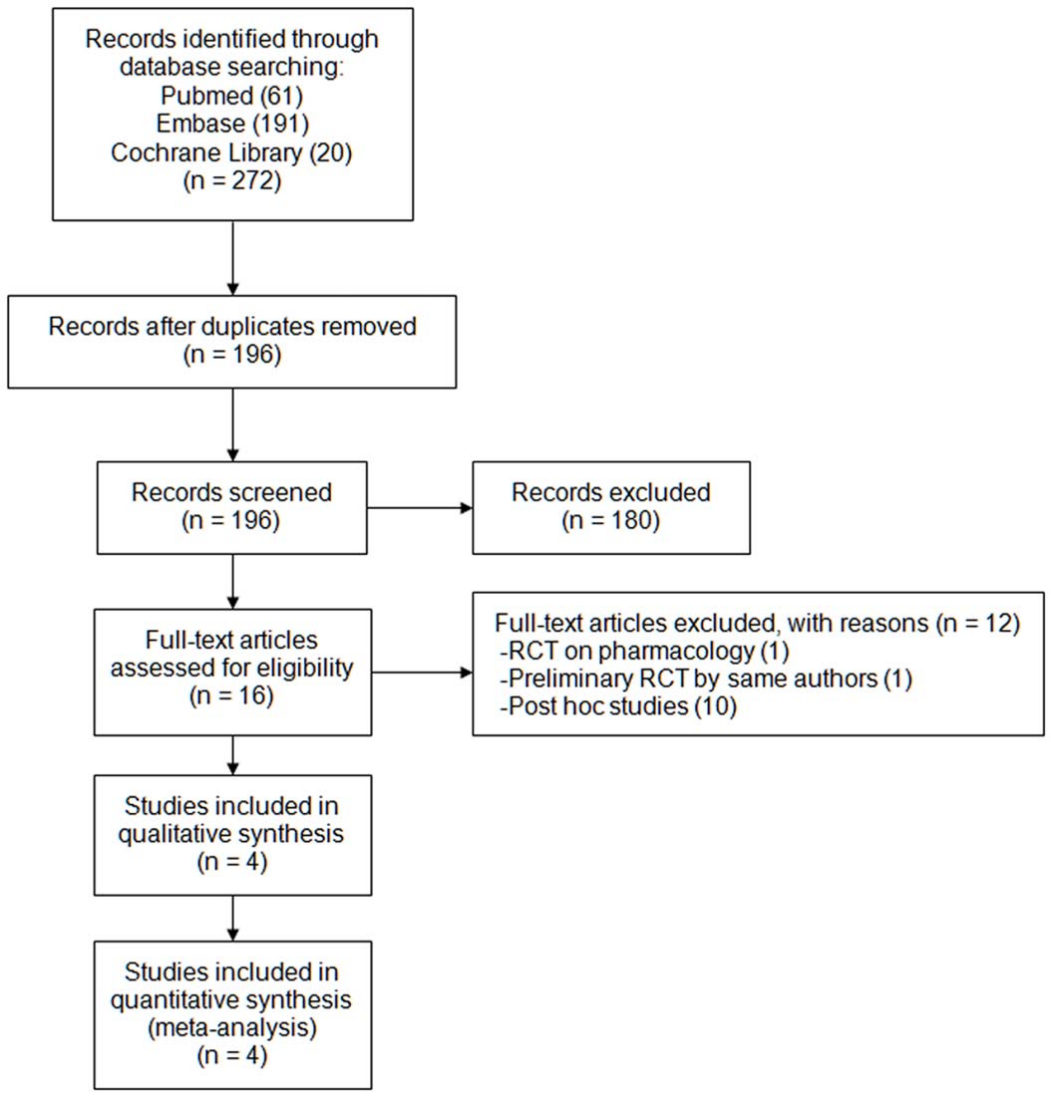
The flowchart shows the selection of studies for meta-analysis.

**Table 1:** Characteristics of RCTs Assessing the Effects of Clazosentan in aSAH and Critical Appraisal of RCTs.

Study/Year	No. of Patients	Age Eligibility, Years	FemaleSex, %	Intervention	Treatment Arms	Treatment Time after aSAH	Treatment Duration	Primary Outcome	Secondary Outcome	Jadad Score	ITT Analysis
Vajkoczy et al. 2005	32	18–65	64.7	Clipping	Clazosentan (0.2 mg/kg/h) vs placebo	<48 h	Max 14d	Angiographic vasospasm	NCI	5	Yes
Macdonald et al. 2008	409	18–71	70.7	Clipping or coiling (185∶224)	Clazosentan (1 mg/h, 5 mg/h, 15 mg/h) vs placebo	<56 h	Max 14d	Angiographic vasospasm	DIND; NCI; Rescue therapy; GOSE; AEs	4*	No
Macdonald et al. 2011	1147	18–75	67.6	Clipping	Clazosentan (5 mg/h) vs placebo	<56 h	Max 14d	Vasospasm-relatedM/M	GOSE	5	Yes
Macdonald et al. 2012	571	19–76	69.9	Coiling	Clazosentan (5 mg/h,15 mg/h) vs placebo	<56 h	Max 14d	Vasospasm-relatedM/M	GOSE	5	No

* Randomization method was not described.

Abbreviations: AEs, adverse effects; DIND, Delayed ischemic neurological deficit; GOSE, Glasgow Outcome Scores Extended; ITT, intention-to-treat; M/M, morbidity/mortality; NCI, new cerebral infarction.

### Selection Criteria

Studies meeting the following criteria were selected: randomized controlled trial; patients with aSAH; treatment of clazosentan compared with placebo, randomly assigned following aSAH; outcomes were reported as vasospasm, vasospasm-related complications, or the functional outcome. In case of multiple reports on the same trial, the one with more comprehensive information was selected for meta-analysis.

**Figure 2:**
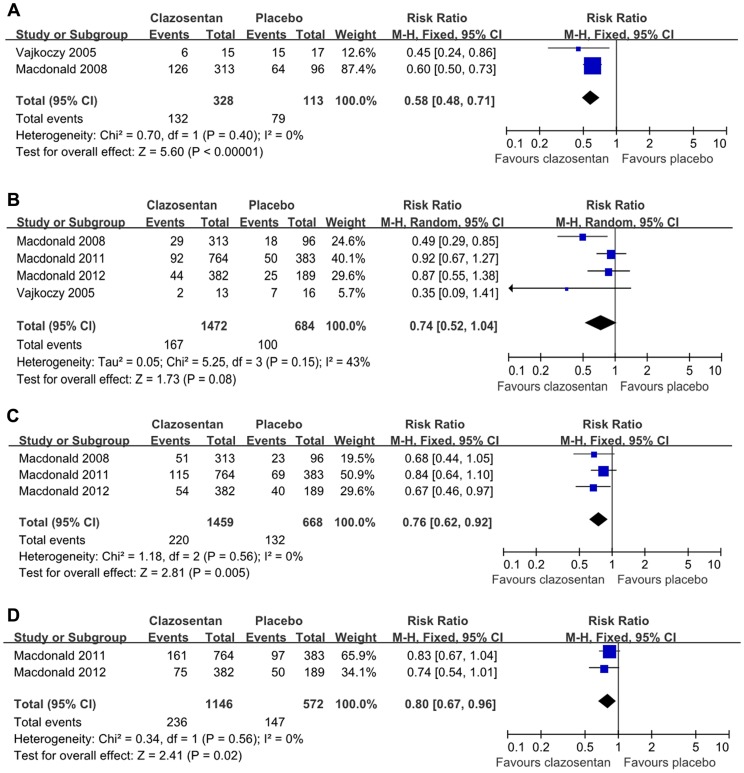
Effects of clazosentan in the prevention of angiographic vasospasm, vasospasm-related NCI, DIND, and vasospasm-related M/M. (A) Forest plot of RR and 95% CI for the incidence of angiographic vasospasm in patients assigned to clazosentan treatment versus placebo. (B) Forest plot of RR and 95% CI for the incidence ofvasospasm-related NCI in patients assigned to clazosentan treatment versus placebo. (C) Forest plot of RR and 95% CI for the incidence of vasospasm-related DIND in patients assigned to clazosentan treatment versus placebo. (D) Forest plot of RR and 95% CI for the incidence of vasospasm-related M/M in patients assigned to clazosentan treatment versus placebo.

### Data Extraction

Two assessors (XWand YML) independently reviewed the full manuscripts of eligible studies. Data were extracted independently in standardized data-collection forms. Extracted data included first author’s name; year of publication; sample size; patients’ characteristics (mean age, gender); surgical intervention; treatment arms; dose; starting time of treatment; treatment duration; rescue therapy; adverse effects; primary and secondary outcomes. Any discrepancy was resolved by discussion or a third author (CGH). Selected RCTs were critically appraised using the Jadad scale, which scores studies’ description of randomization (2 points), blinding (2 points) and attrition information (1 point) [9]. We also assessed whether studies adequately reported concealment of treatment allocation or intention-to-treat (ITT) analysis.

**Figure 3:**
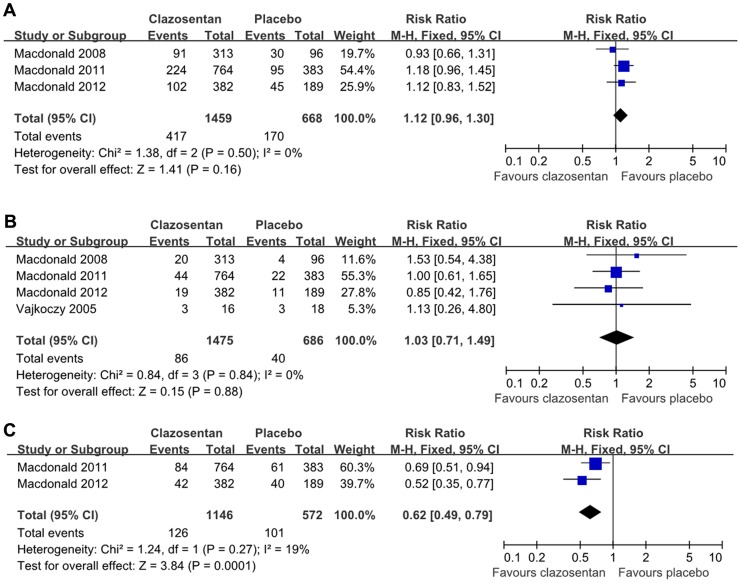
Effects of clazosentan in reducing the occurrence of all-cause death, functional outcome, and the rescue therapy. (A) Forest plot of RR and 95% CI for the occurrence of all-cause mortality in patients assigned to clazosentan treatment versus placebo.(B) Forest plot of RR and 95% CI for the occurrence of poor functional outcome in patients assigned to clazosentan treatment versus placebo.(C) Forest plot of RR and 95% CI for the prevalence of rescue therapy in patients assigned to clazosentan treatment versus placebo.

### Study Outcomes

The outcomes of interest included the occurrence of vasospasm, new cerebral infarction (NCI), DIND, and vasospasm-related morbidity and mortality (MM), which represented the primary outcomes. The secondary outcomes were indicated by all-cause mortality and the functional outcome. Vasospasm was defined as focal or generalized reduction of the arterial caliber in cerebral angiogram (angiographic vasospasm). The term “delayed cerebral ischemia (DCI)” was abandoned due to controversial definitions. Cerebral infarction and clinical deterioration due to DCI have been suggested to be respectively assessed [10], [11]. Accordingly, we predefined vasospasm-related new cerebral infarction (NCI), detected by the postoperative CT scan, as one of the primary outcomes. DIND was defined as a decrease of ≥2 points in the modified Glasgow Coma Scale (GCS) or an increase of ≥2 points in the abbreviated National Institutes of Health stroke scale (NIHSS) lasting for at least 2 h. For patients in whom neurological scales were not assessable, DIND was defined as administration of rescue therapy. Rescue therapy included initiation or increase in dose of an intravenous vasopressor with or without fluid therapy, or intra-arterial vasodilator, or balloon angioplasty. Vasospasm-related M/M was defined by at least one of the following events: death, NCI, DIND, and rescue therapy. Poor outcome was defined as scores of 1–4 in the Glasgow Outcome Scale (GOS) [12].

**Figure 4:**
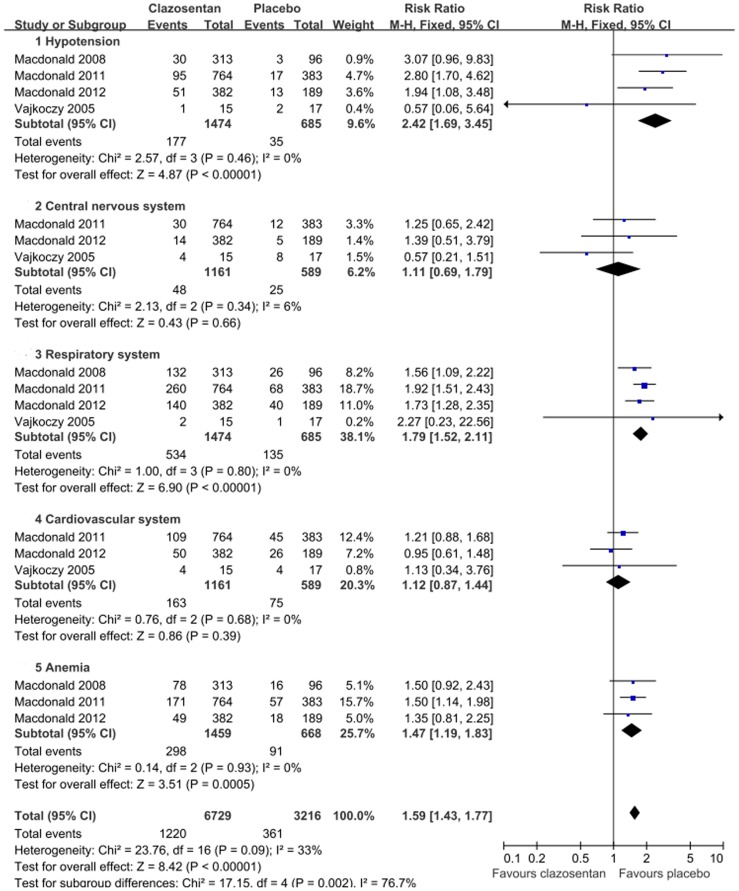
Forest plot shows the incidence of adverse effects in patients assigned to clazosentan versus placebo.

**Figure 5:**
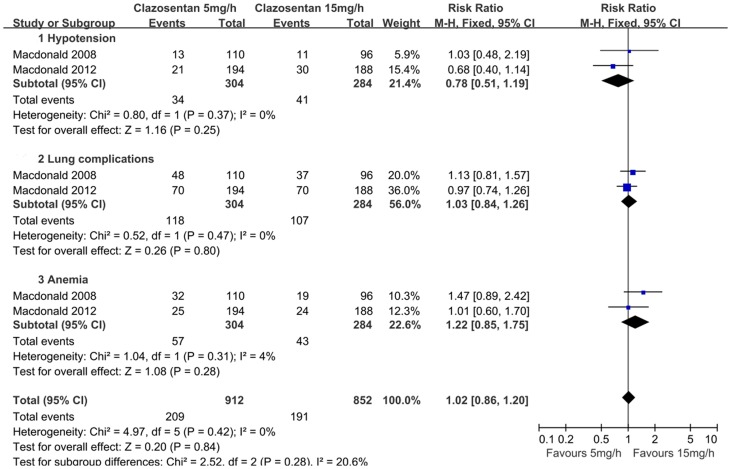
Forest plot on the adverse effects of 5 mg/h and 15 mg/h clazosentan respectively, with subgroup analyses.

### Subgroup Analyses

The subgroup analyses mainly included age, sex, the World Federation of Neurological Surgeons (WFNS) scale, clot size, and dosage of clazosentan, aiming to evaluate their roles in reducing the risk of vasospasm related M/M. Age was trichotomously divided into <50, 50–60, and >60. Sex was dichotomously divided into male and female. The WFNS scale had been widely used to assess the initial clinical and radiologic features of aSAH with 5 grades of severity, which was dichotomously divided into a lower grade (I–II) and a higher grade (III–IV). The higher the score was, the worse the prognosis would be [13].The clot sizes included four types: diffuse thick, diffuse thin, local thick, and local thin. The diffuse thick type was suggested to be one of predominant predictive factors in aSAH [14]. So, it was independently analyzed from other three types. Dosages of the drug were analyzed based on administered doses in different trials.

**Table 2:** Subgroup analyses with pooling RR and 95% CI of CONSCIOUS-2,3 trials.

	RR	95% CI	Heterogeneity test
Sex			
Men	0.82	0.58–1.18	0.574
Women	**0.79**	**0.63–0.99**	0.506
Age (y)			
<50	**0.72**	**0.52–0.99**	0.726
50–60	0.87	0.62–1.20	0.225
>60	0.80	0.55–1.15	0.818
WFNS			
WFNS I,II*	0.73	0.46–1.18	0.065
WFNS III,IV,V*	0.82	0.53–1.26	0.094
Clot size			
Diffuse thick	**0.75**	**0.61–0.93**	1.000
Non-diffuse	0.78	0.53–1.15	0.271

* Data pooled with random effects analysis due to heterogeneity.

Significant effects are shown in bold.

**Table 3:** RRs and 95% CI for different doses of Clazosentan on therisk of vasospasmrelated M/M.

Dose (mg/h)	Trials	Clazosentan			Placebo			RR	95% CI	Heterogeneity
		Events	n	%	Events	n	%			
1	1	39	105	37	36	92	39%	0.95	0.66–1.36	0.77
5	3	238	1064	22.4	184	664	27.7	**0.83**	**0.70–0.98**	0.72
15	2	293	1345	21.8	271	945	28.7	**0.77**	**0.67–0.89**	0.43

Significant effects are shown in bold.

### Statistical Analysis

All outcome variables were dichotomous, as well as the reported AE variables. Review Manager 5.1.7 (Cochrane Collaboration, 2012) was used to process these data. Data with only relative risk (RR) and 95% CI extracted were pooled with Stata 12.0 (Stata Corporation, College Station, TX, USA). RR estimates were calculated to show the effect sizes, with either fixed-effects model or random-effects model used. *P*>0.10 in the 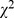 test and I^2^<25% were interpreted as low-level heterogeneity. When significant heterogeneity was absent, the fixed-effects model was employed; otherwise, the random-effects model was used. The statistical uncertainty was expressed in 95% CIs. The publication bias was assessed through funnel plots. A two-tailed *P*<0.05 was deemed statistically significant.

## Results

### Characteristics of Selected Studies

From the 272 initial records, 16 relevant articles were found. Further, 12 studies were excluded, including one preliminary RCT by the same investigators [3], one pharmacological RCT [15], and 10 post-hoc studies of CONSCIOUS-1 trial. 4 RCTs were included in the meta-analysis, with 2156 patients totally (1472 with clazosentan, 684 with placebo) (Figure 1) [4], [5], [6], [7]. The characteristics of these studies were listed in Table 1. All RCTs were of high methodological quality with a satisfying Jadad score. 2 studies were performed on an ITT basis (Table 1) [4], [7].

### Primary Outcomes

#### Angiographic vasospasm

Data of the occurrence of angiographic vasospasm were available in 2 trials, with a total of 441 patients (328 with clazosentan, 113 with placebo) [4], [5]. The occurrence of angiographic vasospasm was significantly reduced in the clazosentan group compared with the placebo one(RR = 0.58; 95% CI, 0.48 to 0.71; *P*<0.05; Figure 2A). The number needed to treat (NNT) for this outcome was 3.4. Data did not indicate heterogeneity (*P* = 0.40).

#### NCI

NCI was reported in all trials, with a total of 2156 patients (1472 with clazosentan, 684 with placebo) [4], [5], [6], [7]. Heterogeneity was observed (*P* = 0.15) and thus the random-effects model was employed. The prevalence of NCI was reduced in the clazosentan group compared with that in the placebo group (RR = 0.74; 95% CI, 0.52 to 1.04, Figure 2B). The NNT was 30.6. However, statistically significance was not observed (*P*>0.05).

#### DIND

DIND was reported in the CONSCIOUS trials, with a total of 2127 patients (1459 with clazosentan, 668 with placebo) [5], [6], [7]. The occurrence of DIND was significantly reduced in the clazosentan group than that in the placebo group (RR = 0.76; 95% CI, 0.62 to 0.92; *P*<0.05; Figure 2C). The NNT was 21.4. Significant heterogeneity was not revealed (*P* = 0.56).

#### Vasospasm-related morbility/mortality

The overall vasospasm-related M/M was assessed in 2 trials, with a total of 1718 patients (1146 with clazosentan, 572 with placebo) [6], [7]. Patients treated with clazosentan had a statistically significant reduction in the occurrence of vasospasm-related M/M (RR = 0.80; 95% CI, 0.67 to 0.96; *P*<0.05; Figure 2D). The NNT was 19.6. Significant heterogeneity was not revealed (*P* = 0.56).

### Secondary Outcomes

#### Functional outcome

In the analyses of poor outcome, 3 CONSCIOUS trials were included [5], [6], [7]. Poor outcome was accordantly defined as GOSE≤4 at week 12. Totally, 2127 patients were available (1459 with clazosentan, 668 with placebo). The overall RR of clazosentan therapy for poor outcome was 1.12 (95% CI, 0.96 to 1.30; Figure 3A). Heterogeneity was not significant (*P* = 0.50).

#### Mortality

All trials reported all-cause death after aSAH [4], [5], [6], [7]. Data were available for 2161 patients (86/1475 died in the clazosentan group, 40/686 died in the placebo group). There was no significant difference between the two groups (RR = 1.03; 95% CI, 0.71 to 1.49; *P*>0.05; Figure 3B). Significant heterogeneity was not observed (*P* = 0.84).

#### Rescue therapy

Rescue therapy was investigated in the CONSCIOUS-2, 3 trials, 1718 patients totally (1146 with clazosentan, 572 with placebo) [6], [7]. Patients treated with clazosentan had a reduced occurrence of rescue therapy (RR = 0.62; 95% CI, 0.49 to 0.79; *P*<0.01; Figure 3C). Significant heterogeneity was not found (*P* = 0.27).

### Safety

TheAEs of clazosentan were listed in all studies, which mainly included hypotension, lung complications, cardiovascular complications, and anemia. Overall, the clazosentan group had a statistically significant increase in AEs compared with the placebo group (RR = 1.59; 95% CI, 1.43 to 1.77; *P*<0.01; Figure 4). A dose-dependent risk of AEs was not observed (Figure 5).

### Subgroup Analyses

Data of subgroup analyses were available in CONSCIOUS-2, 3 trials, with only RR and 95% CI extracted. They were pooled with Stata software. It revealed that age<50, female, and diffuse thick clot had statistically significances in decreasing the incidence of vasospasm related M/M (Table 2). The CONSCIOUS-1,3 trial compared clazosentan in different doses with placebo in the occurrence of vasospasm-related M/M. Pooling data suggested that clazosentan in 1 mg/h had no significant reduction in vasospasm-related M/M, while when the dose was in 5 mg/h or 15 mg/h, statistically significant decreasing was observed (Table 3). The NNT in 5 mg/h and 15 mg/h group were 18.7 and 8.8 respectively.

In our assessment of the funnel plot of each meta-analysis, no evidence for publication bias was indicated.

## Discussion

Since its first isolation from cultured porcine aortic endothelial cells, the critical role of ET-1 in vasospasm has been well-acknowledged, with several ET-receptor antagonists investigated [16]. Kramer et al [17] have conducted a meta-analysis of ET-receptor antagonists in aSAH, including another mixed ET_A/B_ antagonist TAK-004, which was a nonselective ET-receptor antagonist that could diminish the magnitude of ET-1 blockade. The ET_B_ receptor antagonist had been inferred of limited value for vasospasm [16], [18]. Besides, the CONSCIOUS-2,3 trials were not included.

The main findings of our meta-analysis were as follows: First, clazosentan was superior to placebo in reducing the occurrence of angiographic cerebral vasospasm, vasospasm-related DIND, and vasospasm related M/M. Second, no evidence was revealed in the benefits of clazosentan in reducing NCI, overall mortality or improving functional outcome. Third, placebo group had a significant increasing inrescue therapy. Fourth, clazosentan had more AEs compared with placebo, mainly including hypotension, lung complications, and anemia. Fifth, clazosentan in 5 mg/h and 15 mg/h were both effective in reducing vasospasm related M/M, andthe latter had a more favorable NNT. Additionally, AEs were comparable in different doses. Sixth, women, those <50 years of age, and those with diffuse thick clot appeared to have a greater reduction in the risk of vasospasm related M/M with clazosentan.

Several factors support the validity of our meta-analysis. The included RCTs were of satisfying Jadad scores. They were generally comparable with the respect to study design and selection of patients, andthe prognostic baseline characteristics were well-balanced. The timing and duration of clazosentan were similar. Besides, similar criteria of outcomes were implemented. However, several limitations of our study have to be acknowledged. The number of the trials included might be relatively small, with 3 of the 4 analyzed studies designed and implemented by one group, thus decreasing the heterogeneity of the data sources while increasing the internal validity. The sample size, ranging from 32 to 1147, might be too small to show a statistically significant effect on clinical outcome. It has been estimated that more than 5,000 patients are needed to show a treatment effect size of 50% on the mRS 3 months after aSAH [19]. The small amount of patients may render the studies a “failure of randomization”, in terms of imbalance between various prognostic factors. For example, females nearly doubled males in all the trials. Notably, it has been suggested that females are associated with an increased risk of angiographic vasospasm [20]. Our pooling data suggested that female patients with aSAH had a significant reduction of vasospasm-related M/M, which might be criticized for the imbalanced gender at baseline. Moreover, not all the data we concerned were reported in the trials. For example, only 2 trials reported the incidence of angiographic vasospasm. The earliest trial was absent in the incidence of DIND with incomplete follow-up information [4].

Our meta-analysis showed no effect of clazosentan on reducing NCI, mortality or improving the functional outcome. It is instructive to investigate the potential causes that may explain the discrepancies, except the study limitations listed before. Though it was praiseworthy that the results of DCI were centrally reviewed in CONSCIOUS trials, the decision whether a new hypodensity in CT scan could be attributed to vasospasm could still be subjective. In fact, a post hoc analysis of CONSCIOUS-1 trial suggested that there was considerable interobserver variability in attributing CT hypodensities to vasospasm-related lesions [21]. Several processes other than vasospasm may contribute to DIND and poor outcome, including microthrombolism/embolism, impaired cerebral autoregulation, delayed axonal degeneration, impaired stress response, clusters of spreading depolarizaitions, and perforator occlusion attributed to the surgical procedures of clipping or coiling [22], [23], [24], [25], [26]. The effect of clazosentan has been targeted on large vessel vasospasm, with little known effects on microcirculation. Large-vessel narrowing may not have a major impact on brain circulation in the presence of autoregulatory vasodilation of distal arterioles or in the presence of collateral flow [7], [27]. Besides, clazosentan has been experimentally proved useless in preventing neuronal injury or microthromboembolism [28]. Rescue therapy was more frequently seen in the placebo group, which might provide a positive effect on outcome, thus obscuringthe results [6]. Additionally, given a marked increasing of AEs in the clazosentan group, the therapeutic benefits might be counterbalanced [7]. All the 4 trials permitted the use of oral nimodipine which might interact with clazosentan and masked its benefits [6]. Further more, the functional outcome was measuredby GOSE, which was noted to lack specificity in detecting subtle yet meaningful changes in cognition or functioning. In fact, a post hoc analysis has included the scales related to quality of life in patients with aSAH, demonstrating that vasospasm was associated with poor cognition and greater inpatienthealthcare resource use [29].

Dose, AEs, and timing of clazosentan remain subjects of intense debate. Clazosentan in 15 mg/h seemed more favorable in reducing vasospasm related M/M compared with lower dose. Additionally, there was no statistically difference in theoccurrence of AEs in different doses. Compared with placebo, clazosentan was with an increased risk of AEs. However, these AEs, were not only commonly seen in all patients with aSAH, but also manageable and not considered serious [5], [13], [16]. Moreover, although clazosentan was administrated within 56 h up to 14 days after aSAH in all the trials, it has not been validated yet whether it was used timely. On one hand, considering cerebral vasospasm occurs most frequently 7 to 10 days after aneurysm rupture [1], a late use with a shorter course (7–10 d) might be more effective with the decreasing of AEs [14]. On theother hand, it was argued that clazosentan might work better if administered in the first hours after aSAH, given the effect on reversing acute decreasing cerebral perfusion pressure [30].

### Conclusions

Our pooling data support the view that clazosentan is probably effective in the prevention of angiographic cerebral vasospasm, vasospasm-related DIND, vasospasm related M/M, and rescue therapy. However, current evidences do not support benefits of clazosentan in reducing NCI, all-cause mortality or improving functional outcome. Thus, it should be cautious to translate the effects of clazosentan on vasospasm into a clinical benefit. Given the various limitations in previous studies, further researchesare warranted to investigate the possible reasons why clazosentan may have failed to show better clinical outcomes despite of improving vasospasm.
